# Diagnosis of Patent Foramen Ovale: The Combination of Contrast Transcranial Doppler, Contrast Transthoracic Echocardiography, and Contrast Transesophageal Echocardiography

**DOI:** 10.1155/2020/8701759

**Published:** 2020-02-23

**Authors:** Xiaoxue Yang, Hua Wang, Yajuan Wei, Nina Zhai, Baomin Liu, Xiaopeng Li

**Affiliations:** ^1^Department of Ultrasound, The Second Affiliated Hospital of Xi'an Jiaotong University, Xi'an 710004, China; ^2^Department of Ultrasound, Northwest Women and Children Hospital, Xi'an 710061, China; ^3^Transcranial Doppler Sonography Laboratory, The Second Affiliated Hospital of Xi'an Jiaotong University, Xi'an 710004, China

## Abstract

**Objectives:**

To access the distinct values of contrast transcranial Doppler (cTCD), contrast transthoracic echocardiography (cTTE), and contrast transesophageal echocardiography (cTEE) in the diagnosis of right-to-left shunt (RLS) due to patent foramen ovale (PFO) and to define the most practical strategy for the diagnosis of PFO.

**Methods:**

102 patients with a high clinical suspicion for PFO had simultaneous cTCD, cTTE, and cTEE performed. The agitated saline mixed with blood was used to detect right-to-left shunt (RLS).

**Results:**

In all 102 patients, the shunt was detected at rest by cTCD in 60.78% of cases, by cTTE in 42.16%, and by cTEE in 47.06%. The positive results of all 3 techniques with Valsalva maneuver (VM) were significantly improved. cTCD showed higher pick-up rate than cTTE (98.04% vs. 89.22%; *χ*^2^ = 12.452, *p* < 0.05) and the cTEE (98.04% vs. 96.08%; nonsignificant difference) in the diagnosis of PFO. Nevertheless, cTEE, compared with cTTE, underestimated shunting in 44% of patients. The diameter of both PFO entrance and exit was significantly greater in patients with a severe shunt compared with a mild shunt (2.8 ± 1.0 mm vs. 2.0 ± 0.7 mm, *t* = 3.135, *p* < 0.05) and the cTEE (98.04% vs. 96.08%; nonsignificant difference) in the diagnosis of PFO. Nevertheless, cTEE, compared with cTTE, underestimated shunting in 44% of patients. The diameter of both PFO entrance and exit was significantly greater in patients with a severe shunt compared with a mild shunt (2.8 ± 1.0 mm vs. 2.0 ± 0.7 mm, *t* = 3.135, *p* < 0.05) and the cTEE (98.04% vs. 96.08%; nonsignificant difference) in the diagnosis of PFO. Nevertheless, cTEE, compared with cTTE, underestimated shunting in 44% of patients. The diameter of both PFO entrance and exit was significantly greater in patients with a severe shunt compared with a mild shunt (2.8 ± 1.0 mm vs. 2.0 ± 0.7 mm, *t* = 3.135, *p* < 0.05) and the cTEE (98.04% vs. 96.08%; nonsignificant difference) in the diagnosis of PFO. Nevertheless, cTEE, compared with cTTE, underestimated shunting in 44% of patients. The diameter of both PFO entrance and exit was significantly greater in patients with a severe shunt compared with a mild shunt (2.8 ± 1.0 mm vs. 2.0 ± 0.7 mm,

**Conclusions:**

The best method to diagnose PFO should be the combination of cTCD, cTTE, and cTEE. And cTCD should be applied as the first choice for screening RLS. Then, cTTE should be performed to quantify the severity of the shunt. Last but not least, cTEE should be performed to assess the morphologies of PFO when the closure is planned. The study provides for clinicians the most practical strategy for diagnosing PFO in the future. However, further trials with a large sample size are required to confirm this finding.

## 1. Introduction

Patent foramen ovale (PFO) is a remnant of the fetal circulation, which has been also associated with transient ischemic attack (TIA), migraine headaches, unexplained cerebral infarction, decompression illness, and cryptogenic stroke [1, 2]. PFO is more prevalent in young patient suffering from an unexplained cerebrovascular event (<55 years of age) with cryptogenic stroke than in healthy people [3]. It is clinically significant to find a PFO. All contrast transcranial Doppler (cTCD), contrast transthoracic echocardiography (cTTE), and contrast transesophageal echocardiography (cTEE) have been used to detect right-to-left shunt (RLS) due to PFO. Although cTEE has long been considered as the gold standard in the detection of cardiac RLS [4], it frequently provides false-negative results due to sedation for transesophageal echocardiography, which may prevent an adequate Valsalva maneuver (VM) [5]. Klotzsch et al. showed that TEE is more sensitive than TCD (92.3% vs. 84.6%) [6], while Komar et al. found that TCD was greater at diagnosing RLS than TEE [7]. Some studies have reported similar results for the detection of PFO whether comparing TTE with TEE [8] or TCD with TTE [9]. Moreover, a study showed that the closure rate after a PFO closure was more dependent on the anatomy of PFO than on the type of the device [10], so it is clinically significant to assess the PFO morphology prior to percutaneous closure. The most suitable diagnostic strategy for PFO is still controversial. The aim of our study was accessing the distinct values of cTCD, cTTE, and cTEE in the diagnosis of RLS due to patent foramen ovale and to define the most practical strategy for its diagnosis.

## 2. Materials and Methods

### 2.1. Patient Population

We conducted a prospective study that included 102 consecutive patients (40 men and 62 women, mean age: 41.9 ± 13.0[18–71] years, and 85 were <55 years old) with a high clinical suspicion for PFO. Patients who were referred to our neurology department with cryptogenic stroke, transient ischemic attack (TIA), migraine headaches, and unexplained cerebral infarction from December 2017 to May 2019 were enrolled. The study design was approved by the Institutional Clinical Ethics Committee of the Second Affiliated Hospital of Xi'an Jiaotong University and was performed in accordance with the CONSORT 2010 guidelines. And all the patients gave written informed consent. Patients with previous intracranial or extracranial arterial disease and pulmonary arteriovenous abnormalities diagnosed by computed tomography or magnetic resonance imaging or who were unable to perform the VM were ruled out. Baseline characteristics of included patients are summarized in Table 1.

### 2.2. Protocol

All the patients with a high clinical suspicion for PFO were subjected to three examinations. cTCD was performed by a neurologist, and cTTE was performed by an ultrasound technologist on the same day. Then, the patients were subjected to the cTEE examination within a week. The prevalence and severity of RLS-PFO in all patients were analysed by two ultrasound technologists and one neurologist blinded to each other at the time of examination.

During all 3 tests, a mixture of 8 mL of saline, 1 mL of patient's blood, and 1 mL of room air was agitated at least 10 times in 2 10 mL syringes connected by a 3-way stopcock to achieve a good contrast agent [11]. And then the agitated saline solution was vertically injected into the left cubital vein as a bolus both at rest and during VM. All patients had been trained to perform VM before the tests to achieve an intrathoracic pressure of 40 mmHg. We assessed the effective VM by monitoring a peak Doppler flow velocity of the middle cerebral artery reduction > 25% during the cTCD examination or when the transmitral early (E) flow velocity decreases at least 20 cm/sec during the cTTE [12, 13]. For the cTEE tests, the VM was assessed by observing the leftward convexity of the interatrial septum and the crevice size between the atrial septum primum and the atrial septum secundum compared with the normal respiration. The TCD contrast studies were considered positive for PFO when at least 1 microbubble appeared less than 25 seconds after agitated saline injection. We applied a four-level RLS classification based on the high-intensity transient signal (HITS) counts to quantify the degree of shunt, as follows: 0 HITS (negative result); grade I (mild, 1–10 HITS); grade II (moderate, 11–20 HITS); and grade III (severe, >20 HITS or “curtain-like” pattern) [14]. During the cTTE or cTEE studies, a PFO was diagnosed when microbubbles were seen in the left chambers within three cardiac cycles after the opacification of the right chambers. For the cTTE, the extent of the shunt was semiquantified as follows: 0 microbubble (negative result); grade I (mild, 1–10 microbubbles); grade II (moderate, 11–30 microbubbles); and grade III (severe, >30 microbubbles or left ventricle nearly filled with microbubbles) [15]. For the cTEE, quantification of the left atrial was assessed as follows: 0 microbubble (negative result); grade I (mild; 1 to 5 microbubbles), grade II (moderate; 6 to 20 microbubbles), or grade III (severe; >20 microbubbles) [16].

### 2.3. Contrast Transcranial Doppler Examination

For the cTCD, we used a Vivid 7 system fitted with a 2 MHz transducer at a depth of 65 mm in all subjects. While the patients were lying supine, a 20-gauge needle was inserted into the left cubital vein before the test and one middle cerebral artery (MCA) with the superior temporal bone window was monitored. The first injection was performed at rest, and the test was repeated after asking the subject to perform a VM, and the MCA Doppler spectrum was recorded for 25 seconds. The agitated saline solution was injected 5 seconds prior to the start of VM maintaining for more than ten seconds [17]. To avoid possible misinterpretation of the Doppler examinations, VM was performed 3 times with a 5-minute interval, and we regarded one in which the most HITS were recorded from the MCA as the final result.

### 2.4. Contrast Transthoracic Echocardiography Examination

The cTTE study was performed using a Philips Epiq7c system fitted with an S5–1 probe (1–5 MHz). The TTE test was routinely performed to rule out cardiac shunt due to other reasons before the injection of mixture solution. The apical four-chamber view was used to record the count of microbubbles continuously. Following recordings in basal condition, it was repeated during a VM. The VM was performed by the patients blowing into a plastic tube connected to the manometer device [15]. We asked patients to carry out the VM a few seconds before the contrast injection and maintain until the right atrium was filled with the contrast agent. If the testing results with or without VM were positive, two more operations were needed to assess its reproducibility. The maximum number of bubbles recorded from the left ventricle was regarded as the ultimate result.

### 2.5. Contrast Transesophageal Echocardiography Examination

**Figure 1:**
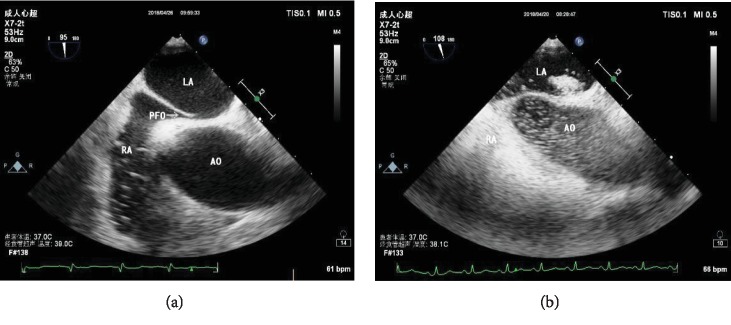
Transesophageal echocardiography showing (a) the morphology of PFO and (b) contrast agent passing through the PFO. LA: left atrium; RA: right atrium; AO: aorta.

A Philips Epiq7c equipment fitted with an X7-2t multiplane probe was used in the cTEE test. All patients were in the left lateral decubitus position and received premedication with oropharyngeal anesthesia to improve tolerance to the test. Before the probe insertion, a spatula was utilized to test its sufficiency. Blood pressure, oxygen saturation, and electrocardiogram were monitored. A comprehensive two-dimensional and colour and spectral Doppler transesophageal echocardiographic examination was performed after insertion of the probe. The area of the atrial septum and the surrounding structures were visualized in the midesophageal bicaval view with an angle of 90° to 120° (Figure 1). While the VM was performed, the ultrasound technologist asked the patients to expand their abdomen and simultaneously pressed the patients' abdomen with a hand to increase the abdomen pressure. The injection of agitated saline was performed a few seconds posterior to the start of VM. We kept continuous recording during basal condition and VM. If the testing results with or without VM were positive, two more injections were needed to assess its reproducibility. The most bubbles seen in the left atrium were regarded as the final result. The maximum diameter of the PFO entrance and exit and the tunnel length were measured during the Valsalva maneuver [10].

### 2.6. Statistical Analysis

Continuous variables were expressed as mean ± standard deviation. Categorical variables were expressed as frequency percentage. We applied the exact McNemar's chi-square test to compare the rates of detecting PFO-RLS (*p* values of <0.05 were considered statistically significant). The two independent samples *t*-test was used to compare mean values for the tunnel length and diameter of PFO on the basis of the different shunts (*p* values of <0.05 were considered statistically significant). The Friedman test was used to analyze the data of the quantification of the shunt (*p* values of <0.05 were considered statistically significant), and then we compared the results of each examination with those by the Wilcoxon matched pairs test (*p* values of <0.0167 were considered statistically significant (with the correction of Bonferroni)). The SPSS 18.0 software package was used for statistical analysis.

## 3. Results

### 3.1. The Pick-Up Rate of PFO-RLS among 3 Techniques

The baseline characteristics of the population included in this study are shown in Table 1. Among the 102 patients who had simultaneous cTCD, cTTE, and cTEE performed, no patient experienced a noticeable adverse event during any procedure or during the 12-hour follow-up period. The pick-up rate of PFO-RLS for all 3 techniques is given in Table 2. The RLS was detected at baseline by cTCD in 62 patients (60.78%), cTEE in 48 (47.06%), and cTTE in just 43 (42.16%). When the VM was performed, the test result was positive in 100 patients (98.04%) in the cTCD examination, in 98 patients (96.08%) in the cTEE examination, and in 91 patients (89.22%) in the cTTE examination. The data revealed that the positive results of all 3 techniques with Valsalva maneuver (VM) was significantly improved and that cTCD showed a higher pick-up rate than cTTE (98.04% vs. 89.22%; *χ*^2^ = 12.452, *p* < 0.05) and cTEE (98.04% vs. 96.08%; nonsignificant difference) in the diagnosis of PFO.

### 3.2. Semiquantitative Shunt Grading

**Figure 2:**
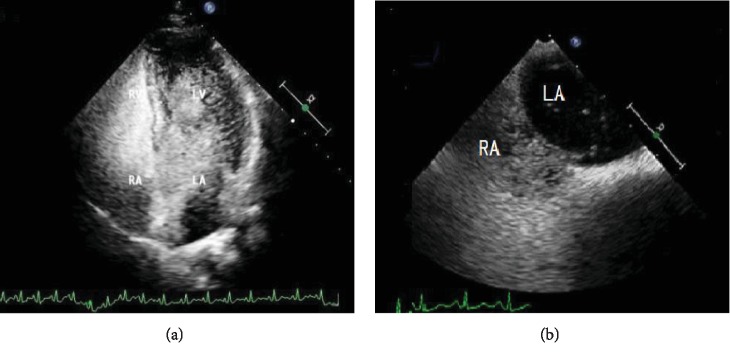
The quantification of the shunt in a patient using two techniques. (a) cTTE shows the left ventricle nearly filled with microbubbles after opacification of the right chambers within the first 3 circles in every single frame image. (b) cTEE shows ≤10 microbubbles appearing in the left atrium after opacification of the right chambers within the first 3 cardiac cycles in every single frame image.

The numbers of cases at each level detected by the 3 techniques are presented in Table 3. It was clear that the 3 techniques yielded different grades in most of the patients, and most disagreement was found for microbubble quantification between grades II and III. In the quantification of shunt, there was a statistically significant difference between the 3 methods (*Z* = 5.887, *p* < 0.05). Shunt was quantified as severe by cTCD in 89 patients (87.25%), cTTE in 76 patients (74.51%), and cTEE in 31 patients (30.39%), and a statistically significant difference was evident from the results between cTCD and cTTE (*p* = 0.003), between cTCD and cTEE (*p* = 0.000), and between cTTE and cTEE (*p* = 0.000). All the above-mentioned data suggested that cTEE obviously underestimated the degree of RLS.  Figure 2 shows that one patient had a severe RLS in the cTTE but mild RLS in the cTEE.

### 3.3. Comparison of the Diameter and Tunnel Length of PFO with Different Shunts

As visualized by TEE, the diameter of both PFO entrance and exit was significantly greater in patients with a severe shunt compared with a mild shunt (*p* < 0.05). There was a nonsignificant difference in tunnel length between patients with mild shunting and severe shunting (*p* > 0.05) (Table 4).

## 4. Discussion

Several studies have reported the usefulness of cTCD, cTTE, and cTEE in the diagnosis of PFO, but a lot of debates remain about whether TCD or TTE could replace TEE as the method for identifying a PFO and what the most suitable diagnostic strategy for PFO is. A meta-analysis showed that TCD had a mean sensitivity of 97% with TEE as the reference standard [18], while another study found that TEE is more sensitive than TCD (92.3% vs. 84.6%) [6]. Some studies have reported similar results for the detection of PFO comparing TTE with TEE [8], but another study reported that cTTE had a low sensitivity (46%) than TEE [19]. In our study, we found that cTCD and cTEE yielded similar results for the detection of PFO, and both of them were more efficacious than cTTE in the detection of RLS. We confirm that cTCD has a good sensitivity in the detection of cardiac RLS since only one patient had a positive result in cTTE and cTEE but had a negative result in cTCD. And cTTE failed to diagnose 7 shunts shown by cTCD and cTEE. We think that there may be several reasons behind the higher pick-up rate of cTCD and cTEE than cTTE. First of all, the ultrasound technologist must visualize the microbubbles during the cTTE and cTEE, but using the automatic counting monitor in cTCD can detect microbubbles that cannot be seen by the ultrasound technologist's naked eye [20]. Secondly, the positive results of RLS in cTCD may include those of the pulmonary arteriovenous fistula and PFO. What is more, the images would shift when performing the VM during cTTE examination. Another limiting factor is the suboptimal quality of images in cTTE due to the patient having pulmonary emphysema or other diseases, which leads to decreased echogenicity. All of these differences may be the major reason for the higher yield of cTCD seen in a lot of studies. However, cTCD by itself does not visualize the anatomic source of the contrast medium [21]. In comparison, cTTE could quantify the severity of the shunt absolutely. Our study showed that cTEE, compared with cTTE, underestimated shunting in 44% of patients. Although the assessment of shunting severity is semiquantitative, several studies suggest that the degree of RLS may correlate with a higher risk for ischemic stroke as well as stroke recurrence [22]. The Valsalva maneuver can increase the degree of shunts and improve the positive results of PFO than normal respiration [16], which was similar to that reported in our study. We found that RLS was detected at rest by cTCD in 62 patients (60.78%), cTEE in 48 (47.06%), and cTTE in just 43 (42.16%). However, the test result was positive in 100 patients (98.04%) in the cTCD examination, in 98 patients (96.08%) in the TEE examination, and in 91 patients (89.22%) in the cTTE examination with VM. Under normal respiration, only few of the PFOs can be detected because the right atrial pressure (RAP) is lower than the left atrial pressure (LAP). When the VM is performed, the sufficient intrathoracic pressure leads to the decrease of venous flow return from the lower and upper extremities and decrease of preload, and it results in RAP to be higher than LAP during the release phase of the VM [23]. Most shunts can be detected with VM due to the inversion of the pressure gradient between the right atria (RA) and left atria (LA). The VM can occur during daily activities, such as lifting heavy loads, defecation, coughing, and vomiting. Therefore, it is important to quantify RLS with VM, which is imperative in all 3 tests when assessing RLS with the agitated saline contrast.

Our study found that cTEE failed to show a RLS in 3 patients with positive results in cTCD and had a tendency to underestimate the severity of RLS. These findings are in concordance with the Van et al. research in which cTEE provided false-negative results in patients with catheter-proven PFOs [24]. One explanation why cTEE might miss RLS is that sedation and esophageal intubation in TEE may weaken the Valsalva maneuver. Another possible reason is the lower intrathoracic pressure in patients performing VM compared with those using a manometer. The Van et al. study [24] found that the right atrial pressures using the manometer 38.7 ± 6.6 mmHg were significantly increased compared with those using VM(21.6 ± 11.9 mmHg, *p* < 0.001). Because the inadequate intrathoracic pressure failed to decrease preload, it cannot achieve the pressure inversion between the right atrium and left atrium during the release phase of VM. In order to overcome this limitation, we pressed the patient's abdomen with a hand while the patient expands his/her abdomen in an attempt to increase abdominal pressure to decrease venous return and preload and subsequently increase the right-atrial-to-left-atrial gradient during the release phase of VM. Although the effectiveness of VM could be enhanced with abdominal compression, compared with an adequately performed Valsalva maneuver, more PFOs were missed. In addition, Johansson et al. indicated that the sensitivity for detection of PFO in cTEE would increase when more injections were given [25]. In our study, if the result was negative in the cTEE test, we would give more injections by up to 4 times. The pick-up rate of PFO-RLS could be improved by multiple injections, and we concluded that RLS would exist as long as there was a positive result in one of the four injections. In conclusion, the cTEE may provide false-negative results for the PFO detection. If the cTEE used in the detection of cardiac RLS solely would miss PFO in some patients, then the subjects could be placed in the wrong categories. So we consider that the combination of cTCD, cTTE, and cTEE should be used to diagnose PFO rather than just cTEE in order to reduce false-negative results. Our study demonstrated that cTEE gave false-negative results and underestimated the shunts in 44% of the patients presenting larger shunts by cTTE. But cTEE has long been considered as the gold standard in the detection of PFO-RLS [4]. It is essential to access the anatomic features of PFO and the surrounding structures such as persisting Eustachian valve, Chiari network, and interatrial septal aneurysm. A PFO closure cannot be safely undertaken without image identification of the location of the lesion. As visualized by TEE, the diameter of the PFO entrance (right atrial margin) is greater than that of the exit (left atrial margin). And we think that high-velocity blood directed by a Eustachian valve from the IVC toward the PFO leads to this result during the release phase of the VM. The diameter of both PFO entrance and exit depends on the shunt severity. Although we found that the tunnel length is not related to the degree of shunt, a study showed that the tunnel length was the most predictive independent factor for residual interatrial shunt at 24 months after technically successful percutaneous PFO closure [10]. So cTEE should be performed to accurately assess the morphologies of PFO when the closure is planned.

Several limitations of this study should be pointed out. Only a single MCA was monitored in our study. If we had monitored bilateral MCA, the cTCD test may yield more positive results. What is more, the contrast agent was injected into the left cubital vein in our study, and contrast-free blood from the inferior vena cava (IVC) sweeps away the contrast, which may have led to insufficient opacification of the right atrium. In this event, femoral vein contrast injection can be applied to avoid this limitation [26]. However, it is not the first choice in clinic to inject contrast into a femoral vein due to its invasiveness. At last, the number of patients in our population is relatively small, and additional prospective studies with larger populations would be desirable.

In conclusion, the best method to diagnose PFO should be the combination of cTCD, cTTE, and cTEE. And we recommend that cTCD should be applied as the first choice for screening RLS owing to its simplicity and inexpensive nature. Then, cTTE should be performed to quantify the severity of the shunt. Last but not least, cTEE should be performed to accurately assess the morphologies of PFO when the closure is planned. This strategy may be helpful in the diagnosis of patent foramen ovale for clinicians in practice.

## Figures and Tables

**Table 1:** Baseline characteristics of the study population.

Clinical characteristics	*N* (%)
Age (year) mean ± standard deviation	41.9 ± 13.0
Sex (male/female)	40/62
Hypertension	15/102 (14.71%)
Hyperlipidemia	8/102 (7.84%)
Diabetic mellitus	12/102 (11.76%)
Coronary heart disease	10/102 (9.80%)
Clinical symptoms	
Transient ischemic attack	24/102 (23.53%)
Cryptogenic stroke	21/102 (20.59%)
Migraine	43/102 (42.16%)
Cerebral infarction	14/102 (13.73%)

**Table 2:** The pick-up rate of PFO-RLS among cTCD, cTTE, and cTEE.

Test method	At baseline	Valsalva maneuver
cTCD	62 (60.78%)	100 (98.04%)
cTTE	43 (42.16%)	91 (89.22%)^†^
cTEE	48 (47.06%)	98 (96.08%)^∗^

PFO: patent foramen ovale; RLS: right-to-left shunt; cTCD: contrast transcranial Doppler; cTTE: contrast transthoracic echocardiography; cTEE: contrast transesophageal echocardiography. ^†^*p* < 0.05 compared with cTCD. ^∗^*p* > 0.05 compared with cTCD.

**Table 3:** The semiquantitative grading of the shunt using 3 techniques.

Grade ^∗^	cTCD	cTTE	cTEE
Negative	2	11	4
Mild	4	4	23
Moderate	7	11	44
Severe	89	76	31

cTCD: contrast transcranial Doppler; cTTE: contrast transthoracic echocardiography; cTEE: contrast transesophageal echocardiography. ^∗^See text for definition.

**Table 4:** Comparison of the diameter and tunnel length of PFO with different shunts.

	Mild	Severe	*t*	*p*
The diameter of PFO right entrance (mm)	2.0 ± 0.7	2.8 ± 1.0	3.135	<0.05
The diameter of PFO left exit (mm)	1.6 ± 0.4	2.2 ± 0.7	−2.582	<0.05
The tunnel length of PFO (mm)	9.3 ± 2.7	9.4 ± 2.9	1.358	>0.05

PFO: patent foramen ovale.

## Data Availability

The data used to support the findings of this study are available from the corresponding author upon request.
